# Endobronchial angiofibroma in the aberrant tracheal bronchus presenting as spontaneous pneumomediastinum

**DOI:** 10.1186/s13019-015-0286-x

**Published:** 2015-07-22

**Authors:** Kyung Soo Kim, Young Kyu Moon, Hyun Woo Jeon, Chan Beom Park, Myeong Im Ahn, Kyo Young Lee, Jae Kil Park

**Affiliations:** 1 Department of Thoracic and Cardiovascular Surgery, The Catholic University of Korea, Seoul St. Mary’s Hospital, 222 Banpo-daero, Seocho-gu, Seoul, 137-701 Republic of Korea; 2 Incheon St. Mary’s Hospital, The Catholic University of Korea, Seoul St. Mary’s Hospital, Incheon, Republic of Korea; 3 Department of Radiology, The Catholic University of Korea, Seoul St. Mary’s Hospital, Seoul, Republic of Korea; 4 Department of Hospital Pathology, The Catholic University of Korea, Seoul St. Mary’s Hospital, Seoul, Republic of Korea

**Keywords:** Pneumomediastinum, Endobronchial tumor, Angiofibroma, Tracheal bronchus

## Abstract

**Background:**

Spontaneous pneumomediastinum is a self-limiting benign disease but abnormal bronchial lesions can be rarely found incidentally, and in selected cases will require surgical resection.

**Materials and methods:**

A 38-year-old man presented with a spontaneous pneumomediastinum. Chest computed tomography revealed an incidental linear endobronchial tumour in the aberrant tracheal bronchus. The tumour was removed surgically and diagnosed with a rare benign tumour of endobronchial angiofibroma.

**Conclusions:**

We report a rare case of endobronchial angiofibroma in the aberrant tracheal bronchus which was detected during the evaluation of a spontaneous pneumomediastinum.

## Background

Spontaneous pneumomediastinum is a self-limiting benign disease that can be managed conservatively, with no need for an invasive procedure. However, abnormal bronchial lesions can be found incidentally, and in selected cases will require surgical resection.

## Case presentation

**Fig. 1:**
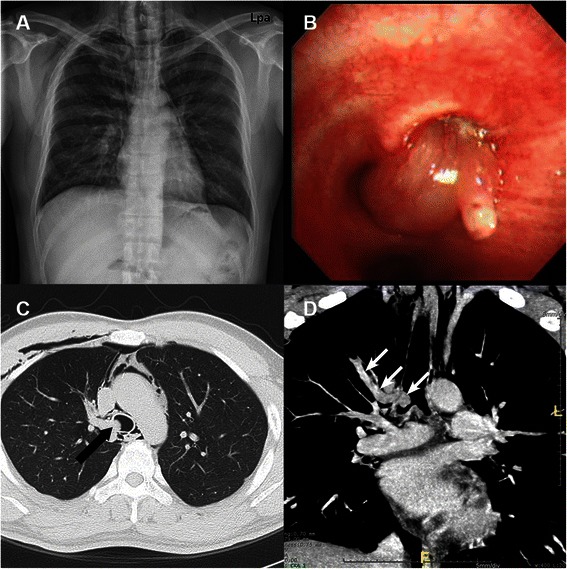
**a** Initial chest X ray shows pneumomediastinum. **b** Bronchoscopy finding of a protruding and glistering tumour originating from the right side of the trachea. **c** Computed tomography showing an elongated endobronchial tumour in the accessory tracheal bronchus originating from the right side of the lower tracheal wall (black arrow, axial view). **d** Computed tomography showing a tumour located in the right upper lobe from the accessory tracheal bronchus (white arrow, coronal view)

A 38-year old male presented at the casualty department with cough, mild dyspnea and neck pain of 1 week duration. A physical examination revealed subcutaneous emphysema on the neck, including the anterior chest wall; the initial chest and neck X-rays revealed a pneumomediastinum (Fig. 1a). The patient denied any recent experience of trauma; yet, he was a current smoker. He was admitted to the hospital and had a conservative treatment. Chest computed tomography for the evaluation of a spontaneous pneumomediastinum disclosed a 7-cm-long, linear mass that was protruding into the trachea. The mass was located in the aberrant tracheal bronchus (Fig. 1c and d). Bronchoscopy demonstrated a protruding and glistering tumour in the aberrant bronchus originating from the right side of the tracheal wall (Fig. 1b), and that the right upper-lobe bronchus, which had a patent orifice, was located in the normal position. An endobronchial biopsy procedure was performed, and the pathology of the removed tissue was found to be suspicious for a leiomyoma.

A muscle-sparing right anterolateral thoracotomy incision was made in the fourth intercostal space. After mediastinal pleural dissection, the right upper lobar pulmonary vessels were ligated and divided. During dissection of the right upper-lobe bronchus, the aberrant bronchus was transected from the trachea and the tracheal portion of the endobronchial mass was readily extracted from the tracheal lumen. The defect in the area of the tracheal lumen was subjected to primary repair with interrupted 4-0 Vicryl sutures (Ethicon, Johnson and Johnson, USA).

**Fig. 2:**
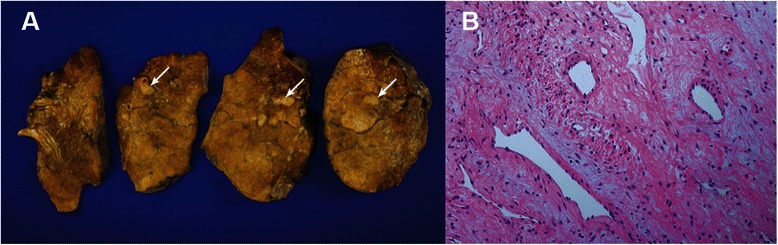
**a** Gross specimen containing a yellowish endobronchial mass in the right upper lobe (arrow). **b** Prominent small-to-medium-sized vessels with hyaline fibrosis in the walls and a complex mixture of stellate and staghorn blood vessels with a background of irregular fibrous stroma (haematoxylin and eosin stain, ×200)

Examination of the gross specimen revealed a yellowish linear endobronchial mass in the aberrant tracheal bronchus (Fig. 2a) and a well-defined endobronchial polypoid mass measuring 5.0 × 1.0 × 0.8 cm. A microscopy examination disclosed prominent small-to-medium-sized vessels with hyaline fibrosis in the walls and a complex mixture of stellate and staghorn blood vessels, with a background of an irregular fibrous stroma (Fig. 2b). In immunohistochemical staining, the spindle cells were stained positively for actin and desmin, and the endothelial cells were stained positively for CD34. The final pathological diagnosis was endobronchial angiofibroma. The patient was discharged on postoperative day 6 with complete resolution of the pneumomediastinum. No tumour recurrence or residual pneumomediastinum was observed during 24 months of outpatient follow-up; furthermore, no other complications occurred.

## Discussion

This is a rare case representing angiofibroma in the aberrant tracheal bronchus that was diagnosed as spontaneous pneumomediastinum during the initial examination. Pneumomediastinum is defined as the presence of air in the mediastinal space, classified in two different types: spontaneous and secondary pneumomediastinum. The pathogenesis of a spontaneous pneumomediastinum is an abrupt increase in alveolar pressure and rupture of the alveoli adjacent to the pulmonary interstitium, thus allowing gas to pass into the mediastinum. Secondary pneumomediastinum is initiated by blunt or penetrating trauma, iatrogenic injury to the esophagus or tracheobronchial tree, rupture of a hollow viscus, or pulmonary or mediastinal infection [1]. The aetiology of the pneumomediastinum in our case is unclear. However, we assumed that the aberrant tracheal bronchus had the “weak point” which was transected during operation. The operative finding of an inadvertent rupture of the aberrant tracheal bronchus and the presentation of repetitive coughing suggests that the pneumomediastinum was caused by minor trauma in the region of the aberrant bronchus as a result of repetitive stress on the tracheobronchial tree induced by coughing. So we thought that there was a causal relationship between the tracheal bronchus and the pneumomediastinum.

Benign tumours of the bronchial tree are very rare (1.9 % of all benign lung tumours); they are asymptomatic when they are small, but often cause localized wheezing, recurrent pneumonia, bronchiectasis and atelectasis as a consequence of bronchial obstruction [2]. A bronchoscopic biopsy followed by complete tumour removal via bronchoscopy and laser therapy, or surgical bronchotomy and lobectomy are the definitive treatment options for endobronchial tumours. A benign endobronchial tumour does not usually recur after a complete resection. Hamartoma, papilloma and leiomyoma are relatively common endobronchial tumours that readily detach from the bronchial wall during removal. However, the literature contains few reports of endobronchial angiofibroma.

The first case of bronchial angiofibroma was reported in a female patient with tuberous sclerosis who had a pulmonary manifestation with multiple white mucosal nodules on the tracheobronchial tree [3]. Endobronchial obstruction by angiofibroma was first described by Early and colleagues, who performed a right middle-lobe lobectomy after locating a 2.5-cm-long and 0.8-cm-diameter polypoid mass in the right middle-lobe bronchus [4]. The endobronchial mass was smooth, pale grey and covered by a uniform layer of benign respiratory epithelium and loose fibrous stroma containing multiple thin-walled blood vessels of various calibres.

Pneumomediastinum associated with the abnormal endobronchial findings is reported only rarely. However, more than 30 % of spontaneous pneumomediastinum are missed in plain X-rays; yet same lesions can be imaged by chest computed tomography [5]. In our case, endobronchial tumor was also detected incidentally by CT scan during evaluation study for spontaneous pneumomediastinum.

## Conclusion

The present case indicates that even though a spontaneous pneumomediastinum is a self-limiting, benign disease that can be managed by a conservative approach, clinicians should consider to exclude secondary pneumomediastinum. In addition, endobronchial angiofibroma in the aberrant tracheal bronchus is rare, but it can be detected during the evaluation of a spontaneous pneumomediastinum, as in the present case.

## Consent

Informed consent was obtained from the patient for publication of this case report and accompanying images. A copy of the written consent is available for review by the Editor-in-Chief of this journal.
